# Probe computing model based on small molecular switch

**DOI:** 10.1186/s12859-019-2767-8

**Published:** 2019-06-10

**Authors:** Yanan Wang, Qi Lv, Yingying Zhang, Luhui Wang, Yafei Dong

**Affiliations:** 1 School of Computer Science, Shaanxi Normal University, Xi’an, 710119 China; 2 College of Life Sciences, Shaanxi Normal University, Xi’an, 710119 China

**Keywords:** DNA molecular computing, DNAzyme, Probe machine, Streptavidin

## Abstract

**Background:**

DNA is a promising candidate for the construction of biological devices due to its unique properties, including structural simplicity, convenient synthesis, high flexibility, and predictable behavior. And DNA has been widely used to construct the advanced logic devices.

**Results:**

Herein, a molecular probe apparatus was constructed based on DNA molecular computing to perform fluorescent quenching and fluorescent signal recovery, with an ’ ON/OFF’ switching function. In this study, firstly, we program the streptavidin-mediated fluorescent quenching apparatus based on short-distance strand migration. The variation of fluorescent signal is acted as output. Then DNAzyme as a switching controller was involved to regulate the fluorescent signal increase. Finally, on this base, a cascade DNA logic gate consists of two logic AND operations was developed to enrich probe machine.

**Conclusion:**

The designed probe computing model can be implemented with readout of fluorescence intensity, and exhibits great potential applications in the field of bioimaging as well as disease diagnosis.

## Background

With the improving complexity of computing problem and limitation of silicon material based microelectronic technology, non-traditional computation methods, such as biological computation, quantum computation, superconducting computation are getting more concerned. Especially, studies on DNA molecular computing, a molecular level data processing, has attracted intense attention in widespread, and scientific studies over the past few decades [1–3]. Unlike traditional digital circuitry whose logic units are physically combined, molecular logic units are functional-combined by associating chemical or biochemical molecules [4, 5].

With the reliable prediction of hybridization behavior based on the Watson-Crick base-pairing principle, DNA is endowed with excellent advantages for the construction of biomolecular computing systems [6–8]. Its simple synthesis method, predictable molecular behavior, and good biological compatibility can largely reduce operating costs, facilitate practical operations in vivo and in vitro, and also provide the potential for the construction of information processing systems [9, 10]. DNA computation is mainly based on the Watson-Crick base pairs principle of DNA molecule to solve problems, DNA sequence is always used as the information carrier, and hybridization reaction is the computation process.

Fluorescent probes can serve as molecular logic gates, as the first report of molecular logic gate by de Silva in 1993 [11]. In the past two decades, excellent work has been devoted to the development of molecular logic gates. Up to now, the fundamental molecular logic gates, such as ‘AND’, ‘OR’, ‘XOR’, and ‘INHIBIT’ [12–14], have been constructed based on synthetic molecules and biomolecules [15–17]. These works not only promote the development of molecular computation but also show great application potential for biomedical imaging [18], data storage [19], medical diagnostics [20], and biochemical analysis [21].

Nucleic acids have emerged as versatile materials due to their unique features of sequence programmability, controllable synthesis and high stability, which allow rational design of complex molecular architectures that are assembled and engineered from defined sequences with high accuracy and precision [22–24]. In dynamic DNA nanotechnology, besides DNA enzyme-activated systems [25–27], strand displacement reaction has demonstrated its powerful capability to build molecular dynamic systems [28]. A typical strand displacement reaction is usually initiated by a short single-stranded overhang region (denoted as a toehold), in which the toehold facilitates the branch migration to displace the resident strand from a double-stranded complex [29–31]. Up to now, strand displacement reactions have been ingeniously implemented in assembly of DNA nanostructures [32–35] and construction of diverse molecular machines [36–38].

In addition, DNAzymes with RNA-cleavage activity have drawn much attention owing to their potential applications in therapeutics[39, 40] and numerous assays [41–43]. The catalytic activity of some DNAzymes is divalent metal ion-specific, similar to the catalytic activity of some protein enzymes that are metal ion cofactor dependent [44, 45]. Moreover, it does not need expert knowledge to design a metal-binding site when compared with organic fluorophores and genetically encoded protein methods, it is also easy to synthesize, modify, and assemble the DNAzymes. And the main feature of the DNA computing device mediated by DNAzyme is that logic operations are mainly realized by hybridization and specifically cleavage. On this basis, a deoxyribozyme is used as a medium for constructing a small molecular switch, in which the cleavage site can be specifically cleaved in the presence of activated deoxyribozyme, with a quenched group-modified short chain is released to generate signal output.

In this work, we proposed a simple probe computing model, which can be implemented by DNA probe technologies based on the strand displacement reaction mediated by streptavidin and DNAzyme. At the first progress, the streptavidin-mediated fluorescent quenching apparatus based on short-distance strand migration was constructed, in which we utilize the DNA strand displacement to manipulate the AND logic operation based on the medium of protein micromolecule-streptavidin, which can highly specifically bound to biotin due to a very strong affinity between them. Then, a signal recovery device triggered by cleave of DNAzyme has been presented. Only when both split DNAzyme segment exits could it have the cleavage activity in the presence of metal ion. To improve the sensitivity and efficiency, we combined streptavidin with DNAzyme to build a cascade logic model which can control sensitively the intensity of signal. At the same time, a small molecular switch that realizing a cascade fluorescent quench and recovery was established.

## Experimental section

**Materials.** All DNA strands were synthesized from Shanghai Sangon and purified by polyacrylamide gel electrophoresis(PAGE) and ULTRA PAGE. The oligonucleotide sequences in the experiment are listed in Table 1.

**Table 1:** Sequences of oligonucleotides used in this work

Oligo name	Sequence(5’-3’)
D	FAM-TTTTTTCTACCAATACTGCTCCGATGATTTTTTTTTT-Biotin
A-f	AGTATTGGTAGA
NAB-f	Biotin-TCATCGGAGCCCTGAACGAC
NAin-f	GTCGTTCAGGaaAGTATTGGTTTTCTCTAT/rA/ggAgCAgTTTTAG
	ATT-BHQ
K-4	TgCggTCTCACATTACTggTgCTgCCTTACgAgTCTTCCACCCATg
	TTAgAgA
M-4	CTgCTCAgCgATgAAgACTCgTAAggCAgCACCAgTAATgTgAgA
	CCg

NAOH, Na_2_EDTA·2H2O, Tris, (HOCH_2_)_3_CNH_2_,CH_3_COOH,C_4_H_6_O_4_Mg·4H_2_O,CH_2_=CH−CONH_2_,(H_2_C=CHCONH)_2_CH_2_,MgCl_2_·6H_2_O,NaCl,(NH4)_2_S_2_O_8_, HCONH_2_, TEMED, (CH_3_)_2_NCH_2_CH_2_N(CH_3_)_2_, 6*loading buffer were bought from Xi’an JingBo Bio-Technique Co. Ethidium bromide (EB), ammonium persulfate, Stain all were bought from Sigma-Aldrich Co. LLC. DNA markers (10 bp ladder and 20 bp ladder) were from TaKaRa biotechnology Co. LTD. TAE/ Mg^2+^ buffer (0.04 ML 1 Tris acetate, 1 mmolL 1 EDTA, 12.5 mmolL 1 Mg acetate, pH 8.0), and the 500 mL mother liquor of acrylamide at the concentration of 40% (217 gacrylamide and 8 g N, N’-Methylenebisacrylamide), 0.5*HEPES buffer (2*HEPES mother liquor, MgCl_2_, 2 M NaCl PH 7.2 ∼7.4).

**Preparation of DNA molecular apparatus.** Before the experiment, various oligonucleotide strand dry powders were dissolved in ultra pure water, and the concentration was measured. Then the amount of each material in the reaction system was calculated based on the measured concentration and the required total volume, and the reaction concentration was generally set to 2 *μ*mol/L. The pH of the solution is strictly controlled between 7.2 and 7.4. Therefore, the pH of the HEPES buffer should be measured with a pH meter before each experiment. If the solution does not meet the pH requirements, it needs to be reconstituted.

The desired buffer and oligonucleotide chain solution were added according to the calculated amount of the reactants, and the mixture was thoroughly mixed to construct a reaction system consist of G1, G2 and ribozyme structures in the model, and they were placed in PCR machine for 2 h.

**Logic Gate Operations.** After constructing the logic gate structure G1, G2 and ribozyme, mixing G1 and G2 in a ratio of 1:1. Then adding a certain amount of streptavidin solution, and standing for 2 h at room temperature in order to fully react. This step is called a DNA strand displacement reaction.

After the strand displacement reaction, the ribozyme is mixed with the solution after the displacement reaction in a ratio of 1:1. And then standing for 4 h at room temperature, the enzyme digestion reaction can be fully reacted. It is called a digestion reaction.

At this point, the model construction reaction in this paper is completed, and then the proposed model will be verified.

**Native PAGE analysis.** The mixture is hybridized under the reaction condition of 95^∘^*C* for 4 min, 65^∘^*C* for 30 min, 50^∘^*C* for 30 min, 37^∘^*C* for 30 min, 22^∘^*C* for 30 min, and 4^∘^*C* for permanent thermal insulation. Then, the obtaining products are detected by native PAGE. The gel was run in 10% acrylamide solution (Acr:Bis =29:1) with 1* TAE buffer, at 100 V constant voltage for 1.5 h. All the gels were run at room temperature and were stained 18 h using Stains-All (Sigma-Aldrich) to image the position of DNA. Photographic images were obtained under visible light with a digital camera.

**Fluorescence measurement.** In this study, we choose to label substrates with fluorophore FAM. And the fluorescent results are obtained using a fluorescent scanning spectrometer for FAM at 492 nm excitation and 518 nm emission by EnSpire ELIASA from PerkinElmer USA.

## Results and discussion

### Streptavidin-mediated fluorescent quenching apparatus

**Fig. 1:**
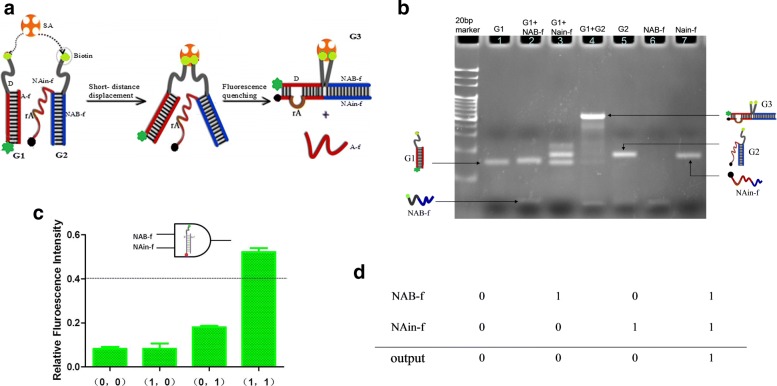
Schematic illustration of “AND” gate A1. **a** The principle of streptavidin mediated fluorescent quenching apparatus based on DNA strand displacement. **b** The PAGE analysis of strand displacing operations (DNA concentration was 1 *μ*M). The gel is dyed by EB. **c** Relative variations of fluorescent intensity. FAM fluorescence was quenching by BHQ, decreasing fluorescent intensity. Here, variations represent the extent of decrease relative to original value. The error bars represent the standard deviations of three independent experiments. The true value table is shown in (**d**)

As shown in Fig. 1a, fluorescent quenching apparatus A1 based on short-distance DNA strand displacement reaction utilizing the specific binding between streptavidin and biotin was proposed to construct a “AND” logic circuit. The double strands G1, as the basic assembly substrate, was formed by partially hybridization of strand D (37nt) and A-f (12nt). Among them, single strand D is modified with fluorophore and biotin at its two terminals, respectively. The inputs are defined as 1 when they are present, whereas as 0 if they are absent. The output is the changes in fluorescence intensity of the system, which is considered as 1 when the fluorescence variation is over 0.4.

Moreover, the polyacrylamide gel electrophoresis (PAGE) images were displaced in Fig. 1b. Initially, without adding any input signals, the substrate G1 is integral and stable, and it has faster gel running speed than other displaced products because of its smaller molecular weight (Fig. 1b, Lane 1). In this experiment, NAB-f and NAin-f serve as two inputs of logic circuit, labeled with biotin and quencher at 5’ of NAB-f and 3’ of NAin-f, respectively. And they can partially hybridize to form the polymer G2 to implement the AND logic operation. When NAB-f and NAin-f are introduced simultaneously, the red part sequence of NAin-f will recognize the complementary regions of D to initiate spontaneous strand migration and displacement based on the combine specifically of streptavidin and biotin, and strand A-f will be released into the solution at the same time. Thereby, quenching fluorophore on the 5’ of D by the quencher on 3’ of NAin-f was occurred and leading to a reduction of fluorescent signal because of the fluorescence resonance energy transfer, with output value 1. And the product G3 will be gradually formed with a slower gel running speed (Fig. 1b, Lane 4). Otherwise, fluorescent quenching based on strand displacement will not occur.

The fluorescence variation was normalized to I=*Δ* F/F_0_, where F_0_ is the fluorescence intensity in the initial state without increasing the input chain; *Δ* F is the change of fluorescence and the average value was obtained by repeating the experiment three times. As shown in Fig. 1c, a significant decrease in fluorescence was almost observed, in the presence of NAB-f and NAin-f. When I_1_= 0.54, which reaching the threshold of 0.4. However, there was less fluorescence reduction with any input added, I_2_= 0.0754 (with NAB-f added) and I_3_= 0.187 (plus NAin-f). It is also worth noticing that the fluorescence change in the presence of NAin-f is relatively high, because there is little binding between NAin-f and D. The fluorescence quenching or the reduction of the fluorescence intensity reaching the threshold represents the output signal 1, otherwise with output value 0. The corresponding true value table is shown in Fig. 1d.

### Apparatus for signal recovery mediated by DNAzyme

Now in order to evaluate the sensitivity and the reversibility of this DNA probe machine model, DNAzyme-assisted double-inputs AND logic model A2 for realizing the recovery of fluorescent signal system was established. Similarly, the inputs are defined as 1 when they are present, whereas as 0 if they are absent. The output is the changes in fluorescence intensity of the system, which is considered as 1 when the fluorescence variation is more than 0.6.

**Fig. 2:**
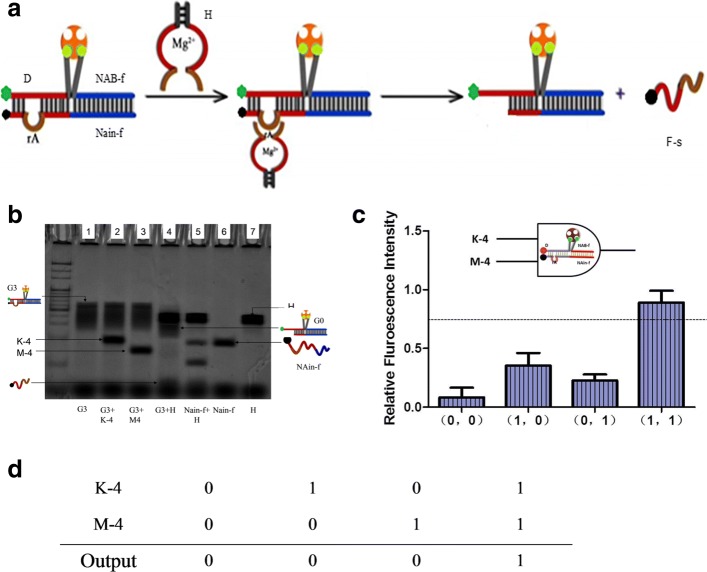
Schematic illustration of “AND” gate A2. The schematic diagram of cleaving reaction mediated by DNAzyme and the principle of signal recovery apparatus are shown in (**a**). For “AND” gate A2, strand K-4 and M-4 are inputs hybridizing to form DNAzme H implementing catalytic cleavage with Mg^2+^, cleaving NAin-f on the brown site. G0 was formed and F-s was released after cleavage, resulting in fluorescent intensity increased as shown in (**c**). There is also the true value table of “AND” logic gate A2 depicted in (**d**). **b** The PAGE analysis of logic operations based on DNAzyme cleavage (DNA concentration was 2 *μ*M, volume 10 *μ*L). The error bars represent the standard deviations of three independent experiments

As is shown in Fig. 2a, the product of the former model G3 is regarded as the substrate of new apparatus, which consists of D, NAB-f and NAin-f, with slower gel running speed (Fig. 2b, Lane 1). The strand K-4 and M-4, including the specific sequence of DANzyme, are two inputs of the device. The double strands H (Fig. 2b, Lane 7), the complete structure of the deoxyribozyme can be formed via hybridization reaction of K-4 and M-4 in the presence of Mg^2+^. Therefore, when K-4 and M-4 are input simultaneously, the K-4 strand hybridizes with M-4 to form H structure, then H was activated to cleave the NAin-f at ribonucleic acid adenosine (rA) site, releasing the short strand F-s (Fig. 2b, Lane 4) labeled with quencher BHQ (Fig. 2a) and producing a fluorescence signal. In Fig. 2b, the lane of H is darker with DNA concentration 2 *μ*M, covering the band of cleaving producer G0 (Fig. 2b, Lane 4 and Lane 7). But the lane of G3 disappeared after adding DNAzyme H, which demonstrates the computing result. In contrast, the initially substrate G3 will not be cleaved in the absence of any input or only one input (Fig. 2b, Lane 1, Lane 2 and Lane 3).

Herein, in this system, in the presence of both K-4 (53nt) and M-4 (48nt), leading to the cleavage of the ribonucleobase(rA)-containing substrate (Fig. 2a) by DNAzyme with Mg^2+^, and output of the AND logic gate is 1, increasing fluorescent intensity. Otherwise, the cleavage-reaction mediated by DNAzyme H will not occurred, with outputting 0. Due to the releasing DNA is short single strand, the gel of electrophoresis in this experiment part is dyed by stain all.

As is shown in Fig. 2c, the fluorescent results were used to further examine the feasibility and effectiveness of the DNAzyme-mediated signal recovery system. Owing to 5’ fluorescence of D and 3’ quencher of NAin-f hybridized for initially stable substrate G3, the fluorescence intensity is very low and even can be ignored. After adding K-4 or M-4, the fluorescence increased by 20.7% and 26.46%, respectively. Compared with the initial state after 8 h, and the result didn’t reaching the preset value. However, it can be observed that addition of two inputs K-4 and M-4 has approximately increasing fluorescent intensity of 81.879%, which strongly demonstrates that the feasibility and validity of the proposed apparatus for the fluorescence signal recovery. The corresponding true value table is shown in Fig. 2d.

### Small molecular switch based on binding induction

**Fig. 3:**
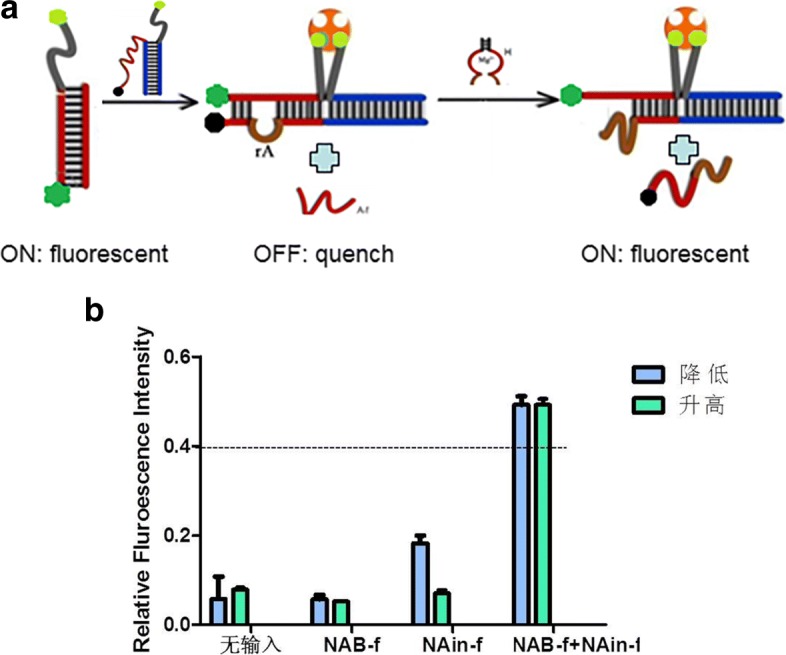
Small molecular switch based on binding induction is shown. **a** The schematic diagram of molecular switch. G1 synthesized by D and NAB-f is the substrate in this system. **b** Relative fluorescent intensity. The blue parts and green parts represent decrease and increment of fluorescent intensity respectively. The error bars represent the standard deviations of three independent experiments

As mentioned above, the streptavidin-mediated AND logic gate A1 achieves fluorescence quenching, while AND logic gate A2 based on DNAzyme catalytic cleavage performs fluorescence recovery. Therefore, to further certificate these operation models, cascading small molecular switch based on binding induction was designed (Fig. 3a). The small molecule switch was measured at the adjustment of G2 and H, and the fluorescence intensity was acted as the output signal, where the high fluorescence intensity was considered as an “ON” state and the low fluorescence intensity was considered as “OFF”.

As is shown in Fig. 3a, firstly, the fluorescently modified D-chain showed high fluorescence which indicating that it was “ON” at this moment. When G2 was introduced, binding-induced strand displacement between G1 and G2 happened because of the specific action of streptavidin and biotin, and strand A-f is released in solution. At the same time, fluorescence was annihilated and it becomes “OFF” state. Then, adding the other regulator DNAzyme H, NAin-f can be effectively recognized and cleaved by H, releasing short strand F-s labeled with quencher (only three base pairs are complementary), resulting in the recovery of fluorescence in the last state “ON”. The results of the relative fluorescent intensity (Fig. 3b) suggested that the molecular switch S is feasible and practicable experimentally from the initial state of “ON” to the second “OFF”, and finally to the last recovering state “ON”.

## Conclusion

In conclusion, a molecular probe apparatus was constructed based on DNA molecular computing to perform fluorescent quenching and fluorescent signal recovery, with an ‘ ON/OFF’ switching function. In our work, the probe computing model have been achieved with biological methods. Fluorescent quenching apparatus based on short-distance DNA strand displacement utilizing the specific binding between streptavidin and biotin were designed to construct a “AND” logic circuit A1 firstly, which implement the turn from high to low of fluorescent intensity. Then, DNAzyme H depending on Mg^2+^ is imported to induce catalytic cleavage of double-input AND operation A2, resulting in fluorescence signal recovery, increasing fluorescent intensity. To validate the sensitivity of the newly developed apparatus, finally, a cascade small molecular switch was constructed to implement decrease and increase of fluorescence in this system. Although there are also some shortages in this work, for example, it is difficult to detect the result of imperfect strand displacement that could result in some incorrect computing conclusions, it could be solved through the continuous optimization of strand sequence and reaction time in subsequent experiment. Compared with other methods, the distinctive advantage of the proposed probe machine is that the streptavidin and DNAzyme are used for molecular computing operation, which provide a new material for logic computing area. Moreover, the advanced probe computing model based on small molecular switch has great potentials in smart bioimaging and disease diagnosis attributed to the biocompatibility of DNA.

## Abbreviations

Co.: Company
EB: Ethidium bromide
PAGE: Polyacrylamide gel electrophoresis
PCR: Polymerase chain reaction
